# Hypophysitis and central nervous system involvement in association with Sjögren’s syndrome along with hypoparathyroidism: a case report

**DOI:** 10.1186/s12883-024-03845-3

**Published:** 2024-09-12

**Authors:** Jungyon Yum, Sang-Won Lee, Yumie Rhee, Kyoung Heo

**Affiliations:** 1 Department of Neurology, Yonsei University College of Medicine, Seoul, Republic of Korea; 2 Division of Rheumatology, Department of Internal Medicine, Yonsei University College of Medicine, Seoul, Republic of Korea; 3 Department of Internal Medicine, Endocrine Research Institute, Yonsei University College of Medicine, Seoul, Republic of Korea

**Keywords:** Hypoparathyroidism, Hypophysitis, Primary Sjögren’s syndrome, Polyautoimmunity

## Abstract

**Background:**

Patients with autoimmune diseases can develop multiple autoimmune diseases over a long period of time, and the presence of more than one autoimmune disease in a single patient is defined as polyautoimmunity. Polyautoimmunity may be clinical evidence that autoimmune diseases share similar immunological mechanisms.

**Case presentation:**

We report a 30-year-old woman with a unique combination of autoimmune diseases predominantly affecting the central nervous system, with hypoparathyroidism, hypophysitis, medulla involvement, and pons and temporal lobe involvement associated with primary Sjögren's syndrome (pSS), occurring independently over a long period. The patient who had a history of muscle cramps and one seizure incident, presented with vomiting and blurred vision. She was diagnosed with hypophysitis and hypoparathyroidism with calcifications in the basal ganglia and cerebellum. She recovered after four months of corticosteroid treatment for hypophysitis and was started on treatment for hypoparathyroidism. Eight months later, she developed vomiting, hiccups, vertigo, and ataxia with a focal lesion in the medulla. She recovered with immunosuppressive treatment for 2 years. Fifty-eight months after the onset of hypophysitis, she developed diplopia and dry mouth and eyes. MRI showed infiltrative lesions in the left pons and left temporal lobe. Based on positive anti-Sjögren's syndrome-related antigen A antibodies and low unstimulated whole salivary flow rate, pSS was diagnosed. She received corticosteroids and continued mycophenolate mofetil treatment with recovery of neurological symptoms.

**Conclusion:**

This case highlights the need for long-term follow-up to detect autoimmune disease processes involving various organs.

## Background

Although autoimmune diseases exhibit contrasting epidemiological features, pathology, and clinical manifestations, these diseases share similar immunogenetic mechanisms (that is, autoimmune tautology) [1]. Therefore, patients with autoimmune diseases have a tendency to develop additional autoimmune diseases [2]. Many of the clusters of autoimmune diseases are well characterized as distinctive syndromes; autoimmune thyroid disease and primary Sjögren's syndrome (pSS) were the most frequent diseases encountered [3]. Some are infrequent and only described in case reports [4]. We report a unique case with hypoparathyroidism, hypophysitis, a focal lesion in the medulla oblongata, and infiltrative lesions in the pons and temporal lobe associated with pSS, occurring independently over a long period of time in contrast to the clinical presentation seen in the previous literature.

## Case presentation

**Fig. 1:**
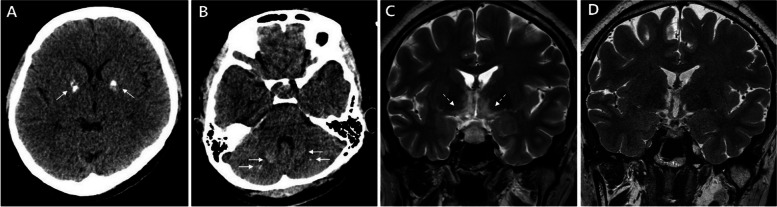
**A**, **B** Non-contrast enhanced CT showing calcifications in the bilateral basal ganglia and cerebellum (arrows). **C** T2-weighted image showing a pituitary mass with infiltrative T2 hyperintense lesions in the hypothalamus, optic chiasm and tracts, and thalamus (dashed arrows). **D** Follow-up MRI performed 8 weeks later showing a marked improvement

A 30-year-old woman presented to our hospital with a one-month history of vomiting and blurred vision. Four years prior, she had a single seizure and subsequently experienced frequent muscle cramps. The patient’s family history was unremarkable, with no history of neck surgery or irradiation. Significant laboratory findings and abnormal hormonal profile were as follows: calcium, 7.6 mg/dL (8.5–10.5); inorganic phosphate, 6.2 mg/dL (2.8–4.5); ionized calcium, 3.86 mg/dL (4.5–5.2); estradiol, < 20 pg/mL (27–433); testosterone, 6.2 ng/dL (8.4–48.1); luteinizing hormone, < 0.2 mIU/mL (1.20–103.03); and parathyroid hormone, 6.6 pg/mL (15–65). A combined pituitary stimulation function test revealed normal pituitary hormone levels. Brain CT scan revealed calcifications in the bilateral basal ganglia and cerebellum (Fig. 1A, B). Hypoparathyroidism treatment (vitamin D, calcium, and calcitriol) was initiated.

Test results for autoantibodies, including anti-aquaporin antibodies, anti-Sjögren's syndrome-related antigen A (SSA), and anti-Sjögren's syndrome-related antigen B (SSB) antibodies, were all negative. Immunoglobulin G4 level was normal. Cerebrospinal fluid (CSF) analysis results were as follows: opening pressure, 265 mmHg; RBC, 5; WBC, 6; protein, 33.7 mg/dL; and glucose, 131 mg/dL. Serum and CSF analysis for infectious etiologies revealed negative results. Analysis of the 22q11 mutation in DiGeorge Syndrome yielded negative results.

Sellar MRI showed a pituitary mass with infiltrative T2 hyperintense lesions involving the hypothalamus and optic chiasm and tracts. Visual symptoms worsened and a follow-up MRI 48 days later showed rapid progression (Fig. 1C). These changes were more consistent with hypophysitis rather than a tumor.

Methylprednisolone pulse therapy (1 g/day) was initiated for 5 days, followed by the administration of prednisolone (60 mg/day), which was tapered and discontinued after 4 months. Visual symptoms improved during treatment. Follow-up MRI showed a significant reduction in the pituitary mass size and a decrease in the extent of T2 hyperintense lesions (Fig. 1D).

**Fig. 2:**
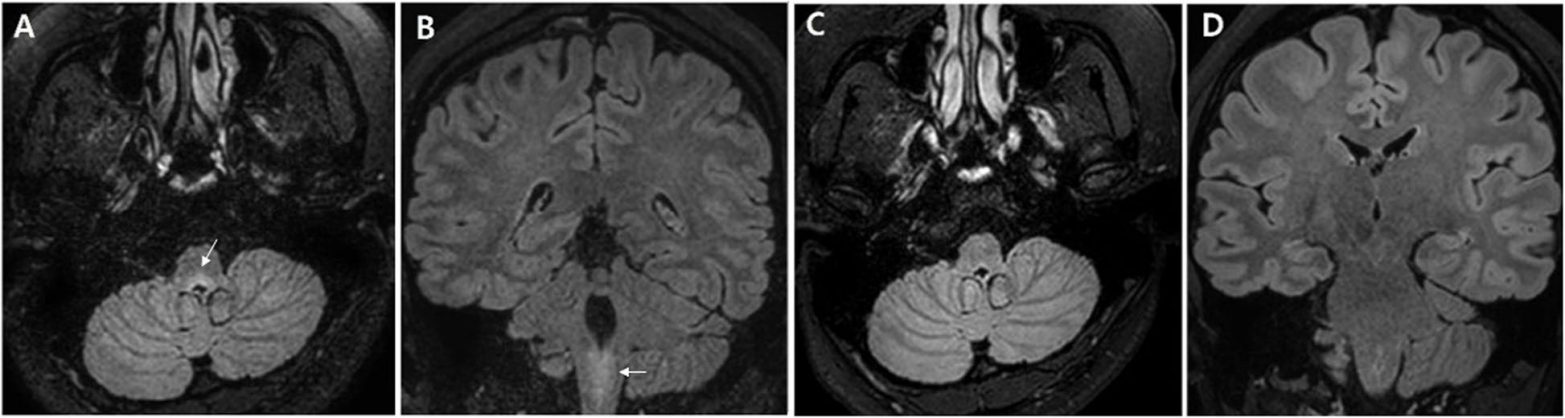
**A**, **B** Fluid attenuated inversion recovery (FLAIR) images showing a focal hyperintense lesion in the medulla adjacent to the foramen of Magendie (arrows). **C**, **D** Follow-up MRI taken 22 weeks later showing a marked improvement

Eight months after the first symptom of hypophysitis, the patient developed nausea, vomiting, and hiccups. Examination revealed up-beating nystagmus and truncal ataxia. Follow-up MRI showed no interval change in the pituitary gland but a focal T2 hyperintense lesion in the medulla adjacent to the foramen of Magendie (Fig. 2A, B). CSF analysis was as follows: opening pressure, 240 mmHg; RBC, 0; WBC, 14; mononuclear cells 100%; protein, 27.9 mg/dL; glucose, 65 mg/dL with serum glucose, 91 mg/dL. CSF cytopathology was negative for malignancies. CSF and serum oligoclonal bands were negative. We initiated corticosteroid therapy with mycophenolate mofetil (MMF). Her symptoms resolved quickly after treatment. A follow-up MRI revealed improved signal changes in the medulla (Fig. 2C and D). MMF was maintained for over 2 years.

**Fig. 3:**
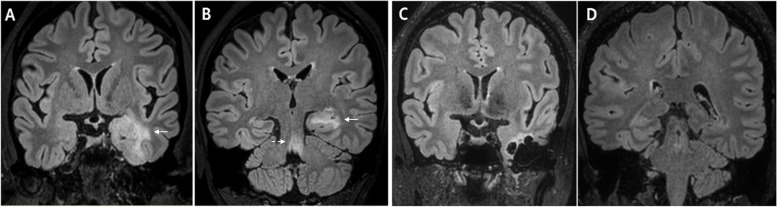
**A**, **B** FLAIR images showing infiltrative hyperintense lesions in the left medial temporal lobe (arrows) and the left dorsal pons (dashed arrow). **C**, **D** Follow-up MRI obtained 2 years showing a marked decrease in the extent of hyperintense lesions with cystic changes related to the biopsy

Fifty-eight months after the onset of hypophysitis, the patient developed diplopia with right medial gaze limitation and gaze-evoked nystagmus, as well as dry mouth and eyes. Anti-SSA antibodies were positive at 22 U/mL (< 7 U/mL). The unstimulated whole salivary flow rate was 0.108 mL/min, just above the classification criteria of ≤ 0.1 mL/min [5]. The Schirmer’s test result was negative. Anti-nuclear antibodies were negative but later became positive (up to 1:640). These findings were appropriate for a diagnosis of pSS. Anti-thyroid peroxidase antibodies were slightly high at 15.5 IU/mL (0–13.7). T3, free T4, and thyroid-stimulating hormone were within normal limits. MRI revealed infiltrative T2 hyperintense lesions in the left medial temporal lobe and the left dorsal pons with no interval change in the pituitary gland (Fig. 3A, B). Temporal lobe lesion biopsy was performed, and the pathological findings were multifocal perivascular lymphocytic infiltration with necrosis and histiocytic infiltration. Corticosteroid therapy was initiated. The nystagmus gradually improved without relapse of neurological symptoms. MMF treatment following corticosteroids has been maintained for 5 years and 6 months with medications for hypoparathyroidism. The dry mouth and eyes persisted. Anti-SSA antibody titers were 68 IU/mL at 1-year, 21 IU/mL at 2-year, and 101 IU/mL at fifty-five months after the first abnormal result. MRI performed 3 months after the third admission showed a marked reduction in the extent of the lesions. MRI performed 2 years after the third admission showed further improvement (Fig. 3C, D).

## Discussion and conclusions

We report a patient with multiple distinct clinical phenotypes arising independently over a long period of time due to autoimmune mechanisms that respond to immunosuppressive therapy.

The most common etiology of hypoparathyroidism is removal or damage of the parathyroid glands during neck surgery, and one of the other causes is an autoimmune disorder [6–8]. Although laboratory data on serum calcium and parathyroid hormone levels were not available at the time of our patient’s symptoms four years ago, the calcifications in the basal ganglia and cerebellum observed on neuroimaging at the first visit strongly suggested the presence of hypoparathyroidism, the first episode of chronic autoimmune disease processes [9].

Hypophysitis is an inflammation of the pituitary gland and it can be primary and idiopathic or autoimmune related, or secondary to local lesions, systemic disease, and medications [10]. Secondary hypophysitis may be associated with autoimmune diseases such as hypoparathyroidism, pSS, and autoimmune polyglandular syndrome (APS) [10–12]. pSS is an autoimmune disease characterized by an autoimmune exocrinopathy involving mainly salivary and lacrimal glands, and may have neurological manifestation. The most common neurological complication of pSS is peripheral neuropathy. The reported neuropathies in pSS included distal sensory polyneuropathy, axonal sensorimotor polyneuropathy, chronic inflammatory demyelinating polyneuropathy, multiple mononeuropathy, sensory neuronopathy, and small fiber neuropathy. Additionally, there have been reports demonstrating the relation of pSS with motor neuron disease and myositis [13]. CNS involvement in pSS is known to be much less common, and the prevalence of CNS involvement in pSS is controversial, ranging from 0 to 68% [14]. CNS involvement in pSS may explained by direct infiltration of the CNS by mononuclear cells, vascular injury related to the presence of antineuronal antibodies and anti-SSA antibodies, or ischemia secondary to small vessel vasculitis [14]. The spectrum of CNS involvement varies with focal central lesions, conditions mimicking multiple sclerosis, encephalitis, aseptic meningitis, cerebellar syndromes causing ataxia, movement disorders affecting the basal ganglia, neuromyelitis optica, problems with memory, cognition, and depression, and rarely hypophysitis [11, 15]. Furthermore, CNS involvement in pSS frequently precedes the diagnosis of pSS [15–17]. Two retrospective studies evaluating patients with pSS found CNS involvement in 5.8% (25/424) and 15% (14/93) of patients, respectively [16, 17]. In both studies, CNS manifestations preceded the diagnosis of pSS in 52% (13/25) and 64% (9/14) of patients, respectively. In our patient, hypophysitis and involvement of the medulla may have occurred as neurological involvement in the subclinical state of pSS. On the other hand, considering that the hypophysitis and medulla involvement occurred fifty-eight and fifty months, respectively, before the diagnosis of pSS, they may have occurred independently of pSS. This is supported by the negative test results for autoantibodies, including anti-SSA and anti-SSB antibodies, and the absence of dry mouth and eyes during that time. One patient with pSS accompanied by hypoparathyroidism was reported to have anti-calcium sensing receptor antibodies. To control active systemic disease associated with pSS, glucocorticoids should be used at the minimum dose and length of time necessary and immunosuppressive agents (cyclophosphamide, azathioprine, methotrexate, leflunomide, and MMF) should be mainly used as glucocorticoid-sparing agents, with no evidence supporting the choice of one agent over another. B-cell targeted therapies (rituximab, abatacept, and belimumab) may be considered in patients with severe, refractory systemic disease [18].

Polyautoimmunity is defined as the presence of more than one autoimmune disease in a single patient. When three or more autoimmune diseases coexist, this condition is called multiple autoimmune syndrome (MAS) [3]. MAS can be classified into three groups according to the prevalence of their associations with one another: type 1, type 2 and type 3. Although pSS is often found in types 2 and 3 MAS, the combination of autoimmune diseases seen in our patient does not fit any of MAS types [2]. The combination of autoimmune diseases can also be called APS. APS is a multifactorial disease characterized by the coexistence of at least two autoimmune-mediated endocrinopathies, which may occur with several non-endocrine autoimmune diseases. APS can be divided into two major subtypes, juvenile and adult, by a specific clustering of monoglandular autoimmune diseases that depends on genetic and non-genetic environmental factors and differs considerably at the time of presentation [19]. Although one endocrinopathy (hypoparathyroidism) observed in our patient does not fit the definition of APS types, hypoparathyroidism is common in the juvenile APS and a rare autoimmune endocrinopathy in the adult APS [19]. pSS and hypoparathyroidism can occur together in the adult APS as seen in our patient [19, 20].

In conclusion, we report a unique case of polyautoimmunity with a very unusual combination of predominant CNS manifestations showing clinical manifestations of hypoparathyroidism, hypophysitis, the medulla involvement, and the pons and temporal lobe involvement associated with pSS, occurring independently over a long period due to autoimmune mechanisms. This case highlights the need for regular long-term follow-up of patients with autoimmune diseases, with close monitoring of a range of symptoms and autoantibodies that may suggest the development of a new autoimmune disease.

## Data Availability

The data used is available from the corresponding author upon reasonable request.

## Abbreviations

pSS: Primary Sjögren’s syndrome
MRI: Magnetic resonance imaging
CT: Computed tomography
SSA: Sjögren’s syndrome-related antigen A
SSB: Sjögren’s syndrome-related antigen B
CSF: Cerebrospinal fluid
MMF: Mycophenolate mofetil
CNS: Central nervous system
APS: Autoimmune polyglandular syndrome
MAS: Multiple autoimmune syndrome

## References

[1] Anaya JM. The autoimmune tautology. Arthritis Res Ther. 2010;12(6):147. 21092150 10.1186/ar3175 PMC3046506

[2] Cojocaru M, Cojocaru IM, Silosi I. Multiple autoimmune syndrome. Maedica (Bucur). 2010;5(2):132–4. 21977137 PMC3150011

[3] Rojas-Villarraga A, Amaya-Amaya J, Rodriguez-Rodriguez A, et al. Introducing polyautoimmunity: secondary autoimmune diseases no longer exist. Autoimmune Dis. 2012;2012:254319. 22454759 10.1155/2012/254319 PMC3290803

[4] Betterle C, Furmaniak J, Sabbadin C, et al. Type 3 autoimmune polyglandular syndrome (APS-3) or type 3 multiple autoimmune syndrome (MAS-3): an expanding galaxy. J Endocrinol Invest. 2023;46(4):643–65. 36609775 10.1007/s40618-022-01994-1

[5] Shiboski CH, Shiboski SC, Seror R, et al. 2016 American College of Rheumatology/European League Against Rheumatism classification criteria for primary Sjogren’s syndrome: a consensus and data-driven methodology involving three international patient cohorts. Ann Rheum Dis. 2017;76(1):9–16. 27789466 10.1136/annrheumdis-2016-210571

[6] Kifor O, McElduff A, LeBoff MS, et al. Activating antibodies to the calcium-sensing receptor in two patients with autoimmune hypoparathyroidism. J Clin Endocrinol Metab. 2004;89(2):548–56. 14764760 10.1210/jc.2003-031054

[7] Harris HE, Kemp EH, Brown EM, Weetman AP, Swaminathan K. First report of anti-calcium-sensing receptor antibodies in a patient with Sjogren’s syndrome and primary hypoparathyroidism. Rheumatology (Oxford). 2011;50(6):1173–5. 21454310 10.1093/rheumatology/ker128

[8] Mannstadt M, Bilezikian JP, Thakker RV, et al. Hypoparathyroidism. Nat Rev Dis Primers. 2017;3(1):17055. 28857066 10.1038/nrdp.2017.55

[9] Zavatta G, Clarke BL. Basal ganglia calcification in hypoparathyroidism and pseudohypoparathyroidism: local and systemic metabolic mechanisms. J Endocrinol Invest. 2021;44(2):245–53. 32661948 10.1007/s40618-020-01355-w

[10] Rawanduzy CA, Winkler-Schwartz A, Couldwell WT. Hypophysitis: defining histopathologic variants and a review of emerging clinical causative entities. Int J Mol Sci. 2023;24(6):5917. 36982990 10.3390/ijms24065917 PMC10057821

[11] Louvet C, Maqdasy S, Tekath M, et al. Infundibuloneurohypophysitis associated with sjogren syndrome successfully treated with mycophenolate mofetil: a case report. Medicine (Baltimore). 2016;95(13):e3132. 27043673 10.1097/MD.0000000000003132 PMC4998534

[12] Bellastella G, Maiorino MI, Bizzarro A, et al. Revisitation of autoimmune hypophysitis: knowledge and uncertainties on pathophysiological and clinical aspects. Pituitary. 2016;19(6):625–42. 27503372 10.1007/s11102-016-0736-z PMC7088540

[13] Perzynska-Mazan J, Maslinska M, Gasik R. Neurological manifestations of primary Sjogren’s syndrome. Reumatologia. 2018;56(2):99–105. 29853725 10.5114/reum.2018.75521 PMC5974632

[14] Tobon GJ, Pers JO, Devauchelle-Pensec V, Youinou P. Neurological disorders in primary Sjogren’s syndrome. Autoimmune Dis. 2012;2012:645967. 22474573 10.1155/2012/645967 PMC3303537

[15] Margaretten M. Neurologic manifestations of primary sjogren syndrome. Rheum Dis Clin North Am. 2017;43(4):519–29. 29061239 10.1016/j.rdc.2017.06.002

[16] Massara A, Bonazza S, Castellino G, et al. Central nervous system involvement in Sjogren’s syndrome: unusual, but not unremarkable–clinical, serological characteristics and outcomes in a large cohort of Italian patients. Rheumatology (Oxford). 2010;49(8):1540–9. 20444860 10.1093/rheumatology/keq111

[17] Moreira I, Teixeira F, Martins Silva A, et al. Frequent involvement of central nervous system in primary Sjogren syndrome. Rheumatol Int. 2015;35(2):289–94. 25056402 10.1007/s00296-014-3097-9

[18] Ramos-Casals M, Brito-Zeron P, Bombardieri S, et al. EULAR recommendations for the management of Sjogren’s syndrome with topical and systemic therapies. Ann Rheum Dis. 2020;79(1):3–18. 31672775 10.1136/annrheumdis-2019-216114

[19] Kahaly GJ, Frommer L. Polyglandular autoimmune syndromes. J Endocrinol Invest. 2018;41(1):91–8. 28819917 10.1007/s40618-017-0740-9

[20] Iizuka K, Mizuno M, Nonomura K, Yabe D. A rare case of autoimmune polyglandular syndrome with Sjogren’s syndrome and primary hypoparathyroidism. BMJ Case Rep. 2019;12(5):e228634. 31133546 10.1136/bcr-2018-228634 PMC6536189

